# First Detection of High-Level Aminoglycoside-Resistant *Klebsiella pneumoniae* and *Enterobacter cloacae* Isolates Due to 16S rRNA Methyltransferases with and Without *bla*_NDM_ in Uruguay

**DOI:** 10.3390/antibiotics13111029

**Published:** 2024-10-31

**Authors:** Romina Papa-Ezdra, Nicolás F. Cordeiro, Federica Ferreira, Virginia García-Fulgueiras, Lucía Araújo, María Inés Mota, Matilde Outeda, Verónica Seija, Rafael Vignoli, Inés Bado

**Affiliations:** 1 Departamento de Bacteriología y Virología, Facultad de Medicina, Instituto de Higiene, Av. Alfredo Navarro 3051, Montevideo 11600, Uruguay; rpapa@higiene.edu.uy (R.P.-E.); ncordeiro@higiene.edu.uy (N.F.C.); fferreira@higiene.edu.uy (F.F.); virginiagarcia@higiene.edu.uy (V.G.-F.); laraujo@higiene.edu.uy (L.A.); imota@higiene.edu.uy (M.I.M.); rvignoli@higiene.edu.uy (R.V.); 2 Laboratorio Central del Hospital Pereira-Rossell, Administración de los Servicios de Salud Estado, Ministerio de Salud Pública, Montevideo 11600, Uruguay; 3 Departamento de Laboratorio de Patología Clínica, Repartición Microbiología, Hospital de Clínicas, Facultad de Medicina, Universidad de la República, Av. Italia s/n, Montevideo 11600, Uruguay; matildeouteda@gmail.com (M.O.); veronicaseija@hc.edu.uy (V.S.)

**Keywords:** 16S ribosomal RNA methylases, *bla*_NDM-5_, IncR

## Abstract

Background: The increase in antimicrobial resistance includes emerging mechanisms such as 16S ribosomal RNA methylases, which confer high-level resistance to aminoglycosides. In this regard, the most predominant genes observed worldwide are *rmtB* and *armA*, but their presence in Uruguay is unknown. Objectives: We describe the genomic characterization of isolates carrying *rmtB* and *rmtC*, together with *bla*_NDM-5_ and *bla*_NDM-1_, respectively, and *rmtD* in our country. Methology: Five isolates from patients admitted to three hospitals were studied. Identification and antibiotic susceptibility testing were performed using the Vitek2 System. Whole Genome Sequencing was conducted, and hybrid assembly was performed with Unicycler. In silico analysis using the Center for Genomic Epidemiology’s tools was undertaken to predict antibiotic resistance determinants, plasmid incompatibility groups, and sequence types. Results: We report three *K. pneumoniae* ST307 isolates with an IncR plasmid carrying *bla*_NDM-5_/*bla*_CTX-M-15_/*bla*_TEM-1B_/*rmtB*/*dfrA14*/*dfrA12*/*sul1*/*qacEΔ1*/*ermB*/*mphA*, one *K. pneumoniae* ST258 harboring an IncC plasmid containing *rmtC*/*bla*_NDM-1_/*bla*_CMY-6_/*aac(6′)-Ib*/*sul1,* and one *E. cloacae* ST88 isolate with an IncFIB/II plasmid hosting *rmtD*, within a novel Tn*21-like* transposon named Tn*7825*, alongside *bla*_OXA-101_/*sul1*/*tet(G)*/*floR*, and a new variant of *bla*_TEM_ assigned as *bla*_TEM-258_. One of the strains, named UH_B2, also carried an IncM1 plasmid encoding *qnrE1*/*bla*_TEM-1_/*bla*_CTX-M-8_ associated with IS*Ecp1*. Conclusions: This is the first description of plasmids harboring 16S rRNA methyltransferases in Uruguay. The association and dissemination of diverse antibiotic-resistant genes underpin the health threat they represent, highlighting the lack of available antibiotics effective against multidrug-resistant microorganisms.

## 1. Introduction

Antimicrobial resistance (AMR) is an evolving threat to public health and modern medicine, challenging the effectiveness of existing treatments [1]. This phenomenon not only compromises current medical practices but also jeopardizes future advances in healthcare. The research and development of new antibacterial agents have not kept pace with the evolution of resistance, leaving patients vulnerable [1].

Moreover, the COVID-19 pandemic has significantly impacted AMR due to the notable increase in length of stay, antimicrobial use, and difficulties in maintaining infection prevention and control measures. As a result, healthcare-associated antimicrobial-resistant infections and deaths in U.S. hospitals rose at least 15% during the first year of the pandemic. Additionally, in 2021, the CDC reported that after years of a consistent decline in healthcare-associated infections, hospitals experienced higher rates in four out of six types of such infections in 2020 [2].

AMR is linked to the Sustainable Development Goals of the World Health Organization (WHO), the Food and Agriculture Organization of the United Nations, the World Organization for Animal Health, and the United Nations Environmental Programme. These international organizations have stated that AMR should be approached from a One Health perspective, highlighting the interrelation between human, animal, and environmental health and acknowledging this problem as one of the major global health challenges of the 21st century [3]. In this scenario, there are bacterial pathogens that serve as indicators of the evolution of AMR. In 2024, the WHO updated the Bacterial Priority Pathogens List to guide the investment in research and development for surveillance and control of antibacterial resistance. The current list includes 15 families of antibiotic-resistant pathogens, categorized as critical, high, and medium priorities for research and development, and public health measures. In this context, Gram-negative bacteria, such as Enterobacterales resistant to third-generation cephalosporins and/or carbapenems, have been listed as critical priority pathogens. This categorization is based on their ability to transfer resistance genes, the severity of the infections and diseases they cause, and their significant global burden, mainly in low and middle-income countries [1].

Carbapenemases are considered the most clinically relevant and epidemiologically significant resistance mechanism in Gram-negative bacteria. These enzymes are globally distributed among various species of Enterobacterales and play a fundamental role in carbapenems’ ineffectiveness, which are often referred to as the “last resort antibiotics” for treating severe infections caused by these pathogens. Carbapenemases are classified, based on their hydrolytic properties and nucleotide sequences, into classes A, B, and D. The most clinically significant enzymes include KPC variants from class A, NDM from class B, and OXA-48 from class D. In Latin America, the most frequently reported class A, B, and D carbapenemases in Enterobacterales are KPC-2, KPC-3, NDM-1, and OXA-48, each exhibiting specific distribution patterns across different countries [4]. For instance, in our country, KPC-2 and NDM-1 have been reported in clinical *Klebsiella pneumoniae* isolates, among other species [5,6].

The increase in AMR also includes emerging resistance mechanisms to broad-spectrum antimicrobials that inhibit protein synthesis in bacteria, such as aminoglycosides. These antibiotics have returned to practical medicine as potential therapeutic options for infections caused by Gram-negative bacteria, particularly carbapenemase-producing Enterobacterales [7]. However, their clinical use has been hampered by the emergence of transferable resistance mechanisms during the past years. Aminoglycoside resistance is usually due to decreased permeability, augmented drug efflux, enzymatic drug modification or inactivation, or either mutation or modification of the aminoglycoside-binding site [8]. Clinically, the most relevant resistance mechanisms are those acquired through mobile genetic elements (e.g., plasmids and transposons) such as aminoglycoside-modifying enzymes (AMEs). These enzymes can be categorized into three classes: those that inactivate aminoglycosides via acetylation (acetyltransferases), adenylation (nucleotidyltransferases), or phosphorylation (phosphotransferases). Another relevant aminoglycoside-resistance mechanism is target site modification via 16S ribosomal RNA methylases (16S-RMTases) [9]. The latter has gained importance on account of conferring high-level resistance to almost all clinically used aminoglycosides.

Chemically, 16S-RMTases catalyze the methylation of specific nucleotides on the 16S subunit of rRNA, thus interfering with aminoglycoside binding to their target. In this regard, N7-G1405 16S-RMTases are the most common plasmid-borne methylases. These enzymes comprise ArmA and 10 RmtA-H variants worldwide, capable of rendering useless all 4,6-disubstituted aminoglycosides (i.e., plazomicin, amikacin, gentamicin, and tobramycin). Conversely, other 16S-RMTase enzymes, such as NpmA, also confer resistance to 4,5-disubstituted and monosubstituted aminoglycosides (i.e., neomycin and apramycin) [9,10].

Among the N7-G1405 16S-RMTases, *armA* and *rmtB* are the most frequently reported genes and are often associated with other resistance genes, such as those conferring resistance to extended-spectrum β-lactamases (*bla*_CTX-M_), and plasmid-mediated quinolone resistance genes (e.g., *qnrA* and *qnrB*) [9,11].

Nevertheless, from a clinical standpoint, the most concerning scenario is the presence of RmtB methylases in carbapenemase-producing Enterobacterales [7,9]. In Brazil, the coexistence of RmtB in KPC-2-producing *Klebsiella pneumoniae* clinical isolates, or ArmA along with KPC-2 or NDM-1, has already been reported [7]. Furthermore, RmtB was also identified in a clinical isolate of NDM-5-producing *Escherichia coli* in Argentina, whereas Delgado-Blas et al. reported the appearance of *rmtE* in Venezuela [12].

Interestingly, in our country, our group already has reported the presence of *rmtC* in *Acinetobacter baumannii* clinical isolates, and *rmtG* in Enterobacterales isolated from fecal samples of one-day-old chickens imported from Brazil [13,14]. However, there is no information regarding the occurrence of 16S-RMTases in Enterobacterales of human origin in our country.

This study describes the first five *Enterobacteriaceae* human isolates harboring 16S-RMTases in Uruguay. Moreover, we describe the plasmids harboring these 16S-RMTases—*rmtB1* and *bla*_NDM-5_, *rmtC* and *bla*_NDM-1_, as well as *rmtD2*—in plasmids belonging to incompatibility groups IncR, IncC, and IncFIIB(k)/FII, respectively.

## 2. Results

### 2.1. Isolates and Antibiotic Susceptibility Testing

Of the 167 isolates analyzed, 44 were resistant to gentamicin and amikacin, and 5 of these passed the screening assay since they were able to grow in MacConkey-lactose agar plates supplemented with 200 mg/L of both aminoglycosides.

Five isolates with high-level aminoglycoside resistance (HLAR) were collected from clinical samples (blood culture, urine culture, abscess) and one rectal colonization sample from patients admitted to “Hospital de Clínicas”, “Hospital Pasteur”, and a pediatric Hospital “Hospital Pediátrico Centro Hospitalario Pereira Rossell” between 2010 and 2023.

The isolates comprised four *Klebsiella pneumoniae* and one *Enterobacter cloacae*. Using PCR-based screening for genes conferring HLAR we detected the presence of *rmtB* in three isolates (labeled UH_B1, UH_B2, and UH_B3), and *rmtC* and *rmtD* in one isolate each (UH_C1 and UH_D1, respectively).

The isolates UH_B1, UH_B2, and UH_B3 were characterized as *K. pneumoniae* ST307, whereas UH_C1 was characterized as *K. pneumoniae* ST258. These isolates also exhibited resistance to carbapenems, and thus, screening for carbapenemases was conducted using the double-disk synergy test with EDTA and boronic acid, of which the results were suggestive of the presence of metallo-β-lactamases. This was confirmed by PCR and subsequent sequencing, showing the variants *bla*_NDM-1_ and *bla*_NDM-5_. Additionally, these strains showed resistance to aztreonam, ceftazidime-avibactam, ciprofloxacin, and trimethoprim-sulfamethoxazole.

On the other hand, isolate UH_D1 was identified as *E. cloacae* ST88, displaying resistance to third-generation cephalosporins, aztreonam, ciprofloxacin, and trimethoprim-sulfamethoxazole, albeit remaining susceptible to cefepime and carbapenems. All isolates were susceptible to colistin and tigecycline (Table 1).

### 2.2. Characterization of rmtB-Carrying Isolates

The UH_B1, UH_B2, and UH_B3 isolates carried *rmtB1* in IncR plasmids (acc. Nos. PQ203016, PQ212794, and PQ203988, respectively). The plasmids from the first two showed a similarity of 100% and spanned 55.9 kb, while the third had a size of 61.3 kb (Table 1).

These plasmids shared the common IncR backbone genes *repB*, *parAB*, *umuCD*, *retA*, and *resD*, whereas the MDR regions were located between the group IIB intron-encoding reverse transcriptase *retA* and the toxin/antitoxin system *vagD*.

**Figure 1:**
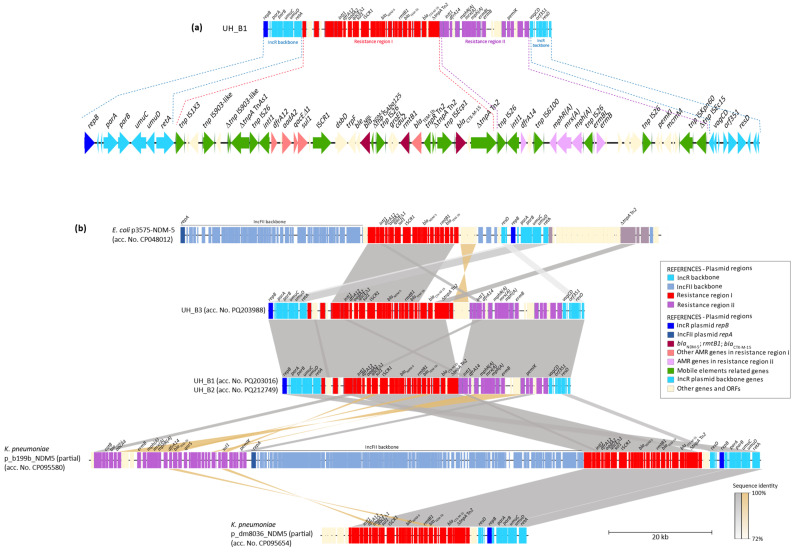
Linear representation of the *rmtB1*-harboring plasmids. (**a**) Linear map of UH_B1 plasmid; (**b**) sequence comparison of the *rmtB1*-harboring plasmids of the *K. pneumoniae* ST307 strains UH_B1 (acc. No. PQ203016), *K. pneumoniae* ST307 UH_B2 (acc. No. PQ212749), and *K. pneumoniae* ST307 UH_B3 (PQ203988), against database retrieved complete (p_3575_NDM-5) or partial (p_b199b_NDM5, p_dm8036_NDM) plasmid sequences. Genes are represented by bars or arrows and colored according to their function or location in the plasmids. Regions were defined for a better understanding of the scheme.

This MDR region harbored *rmtB1* along with *bla*_NDM-5_ in a module flanked by IS*26*, which included the highly conserved environment of *bla*_NDM-5_, composed of a truncated IS*Aba125* on one side and the segment *ble*_MBL_-*trpF*-*tat* in the other. Upstream of this module, a class 1 integron (carrying *dfrA12*-*aadA2*) was associated with IS*CR1*, while downstream, *rmtB1* and *bla*_TEM-1b_ were present, associated with IS*Ecp1* and *bla*_CTX-M-15_. Additionally, there was another class 1 integron (harboring *dfrA14*), although it was truncated by IS*6100*, followed by a module composed of the macrolide-resistance genes *mph(A)* and *ermB* (Figure 1).

BLAST analysis showed similarity with a multireplicon IncFII/IncFII(K)/IncR plasmid named p_b11b_NDM5 from a *K. pneumoniae* isolate (acc. No. CP095580), which harbored both modules, i.e., the one containing *bla*_NDM-5_-*rmtB1*-*bla*_CTX-M-15_, as well as the module containing *dfrA14*-IS*6100*-*mphA*-*ermB*, although in different plasmid regions. Furthermore, another similar IncFII(K)/IncR plasmid (p_dm8036, acc. No. CP095654) was found, albeit without the macrolide-resistance module.

On the other hand, in an *E. coli* isolate carrying the IncFII/IncR p_3575_NDM-5 plasmid (acc. No. CP048012), the module comprising the class 1 integron harboring *dfrA12-aadA2*, *bla*_NDM-5_-*rmtB1-bla*_CTX-M-1-5_ was identical. However, the association with *bla*_CTX-M-15_ or the resistance genes to trimethoprim (*dfrA14*) and macrolides was not observed. Additionally, p_3575_NDM-5 maintains the backbone of IncR plasmids with *repB*-*parAB*-*umuCD* but without the toxin/antitoxin genes *vagCD* (Figure 1).

Although IncR plasmids can be found co-integrated, forming multireplicon plasmids, together with replicons IncC, IncN, IncHI, and InFII backbone regions, the *rmtB1*-carrying plasmids described in our study contain only a single replicon, and no conjugative transfer genes were detected. Nevertheless, *rmtB* and *bla*_NDM-5_ genes were transferred during conjugation assays using isolates UH_B1 and UH_B2 as donors (Figure 1).

In addition to the IncR plasmid, strain UH_B2 also carried two other plasmids. Firstly, an IncM1 plasmid spanning 68,728 bp, which harbored the ESBL gene *bla*_CTX-M-8_, along with IS*26* and the plasmid-mediated quinolone resistance gene *qnrE1*, associated with IS*Ecp1* (accession No. PQ203015). Secondly, an IncFIB(K)/IncFII plasmid of 129,906 bp harboring resistance genes to macrolides (*mphA*), aminoglycosides (*aac(6′)Ib*, *aadA2*, *aac(3)IIa*), trimethoprim (*dfrA12*), chloramphenicol (*catB3*), aminoglycosides fluoroquinolones (*aac(6′)Ib-cr*), and to oxyimino-cephalosporins (*bla*_SHV-28_).

Interestingly, the three *rmtB*-carrying plasmids also harbored anti-phage defensive genes. The *mazEF* toxin/antitoxin system was observed, which prevents the proliferation of phage P1 during *E. coli* infection, as well as a restriction-modification (RM) system, which works as an immune system of sorts against exogenous genomes by using a DNA methyltransferase that modifies a specific sequence motif, thereby protecting its bacterial host DNA from restriction endonucleases, while exogenous DNA remains susceptible to these enzymes.

Genome comparison of the three *rmtB*-harboring isolates showed the occurrence of 11 SNPs. In this regard, and considering the period between the recovery of said isolates, these minor changes suggest that UH_B1, UH_B2, and UH_B3 belong to the same strain.

### 2.3. Characterization of the rmtC-Carrying Isolate

The isolate UH_C1 corresponded to a strain of *K. pneumoniae* from the high-risk clone ST258. It harbored a conjugative plasmid designated as pUYrmtC117, an IncC-type 1 plasmid of 138,998 bp (Genbank PQ122004). pUYrmtC117 carried the *rmtC* and *bla*_NDM-1_ genes as part of the antibiotic resistance island (ARI-A), classically described in IncC plasmids [15]. This region comprised a Tn*1696* transposon with its IRR (IR_tnp_) interrupted by IS*4321*, followed by its respective resolvase and a class 1 integron containing only the aminoglycoside resistance gene *aac(6′)Ib*. Downstream, we detected a DNA methyltransferase and a UV-lyase adjacent to the *rmtC* gene. Further downstream, IS*Kpn14* and *bla*_NDM-1_ were embedded in a truncated Tn*125* within its commonly described genetic background, which includes *ble-trpF*-*dsbD*-*cutA*-*groES*-*groL*-ΔIS*CR27*, followed by the typical Δ*rhs1* of IncC (Table 1). This plasmid also carried an IS*Ecp1*-*bla*_CMY_-derived island (in this case, *bla*_CMY-6_) located between the *traA* gene and *orf1832*. Additionally, and downstream, in the intergenic region between ORFs 82 and 83 (taking as reference plasmid pB2-1, acc. No. KX458222), IS*Kpn8* was not associated with any additional resistance genes. 

Contrary to other IncC plasmids, pUYrmtC117 lacked the ARI-B region (normally located between *dcm* and *parA* genes). Additionally, no phage defense mechanisms were found.

### 2.4. Characterization of the rmtD-Carrying Isolate

On the other hand, *rmtD2* was detected in isolate UD_D1 (characterized as *E. cloacae* ST88), embedded in an IncFIB(K)/IncFII plasmid (designated as pUyrmtD2, acc. No. PQ328755) with a size of 149,643 bp. Although IncF plasmids encode the necessary genes required for mobilization, our attempts at obtaining transconjugants with plasmid pUYrmtD2 yielded negative results.

**Figure 2:**
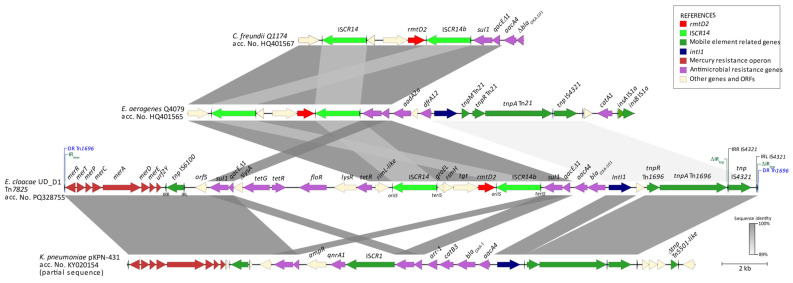
Linear map of Tn*7825* (*E. cloacae* ST88 strain UD_D1) and BLAST comparison results within GenBank available sequences: *C. freundii* Q1174 (acc. No. HQ401567.1), *E. aerogenes* (acc. No. HQ401565.1), and *K. pneumoniae* pKPN-431cz (acc. No. KY020154.1). Genes are represented by arrows and colored according to their function.

The BLAST analysis against the GenBank database showed that the *rmtD2* gene was encoded in a novel Tn*21-like* transposon dubbed Tn*7825* (30,666 bp). This transposon included, adjacent to the IRL (IR_mer_) of Tn*1696*, the heavy metal resistance operon *mer*, along with the insertion sequence IS*6100*. This was followed by tetracycline resistance genes (*tet*), IS*CR14*, *rmtD2*, and the class 1 integron In*14* (i.e., *aacA4*-*bla*_OXA-101_). The 3′ end of Tn*7825* consisted of the *tnpR* and *tnpA* genes of Tn*1696*, in which its IRR (IR_tnp_) was disrupted by the insertion sequence IS*4321*. Similar structures corresponding to the *mer* operon and Tn*1696*, with their IRR disrupted by IS*4321*, have been detected in *K. pneumoniae* isolates in the Czech Republic (accession No. KY020154.1). On the other hand, in Argentina, *rmtD2,* along with IS*CR14* and a class 1 integron containing *aacA4*, has been described in *C. freundii* Q1174 (acc. No. HQ401567.1) and *E. aerogenes* (acc. No. HQ401565.1) (Figure 2).

Interestingly, defensive anti-phage systems were also detected in this plasmid namely, the Cyclic-oligonucleotide-based Anti-phage Signaling System (CBASS), the toxin/antitoxin systems involved in programmed cell death Mok-Hok-Sok and PfiAT, and the two-component defensive system (Detocs) responsible for premature phage lysis.

On the other hand, the UD_D1 isolate harbored a second IncFII plasmid spanning 130,409 bp, which contained a novel β-lactamase, *bla*_TEM-258_ (acc. No. PQ362078). This β-lactamase corresponds to a new allelic variant of *bla*_TEM-2_ as the result of a point mutation at position L67M.

## 3. Discussion

Despite not being a new phenomenon, the emergence of aminoglycoside resistance mediated by 16S rRNA methyltransferases represents an increasing event in the Americas. According to Sellera et al., there has been a significant rise in the reports of these enzymes over the last five years, with a notable increase since 2020 [11].

The information available on the American continent is mainly represented by occurrences in North America (95% of the reports), while the data regarding the rest of the continent is scarce. With this in mind, the most frequently reported genes are *armA*, followed by *rmtF* and *rmtB*. However, the distribution of methylases may differ in the southeastern countries of the continent, where the most commonly detected enzymes are *rmtB*, *rmtC*, *rmtD*, and *rmtG* [11].

In this work, we sought the presence of 16S-RMTases in collections of isolates obtained over the period 2006–2023, and the variants detected were *rmtD2* (in one isolate from 2010), *rmtC* (in one isolate from 2017), and *rmtB1* (in three isolates recovered between 2022 and 2023).

The *rmtD2* gene was reported for the first time in Argentina in 2011 in isolates from collections obtained in the mid-1900s [16]. Nevertheless, only partial conjugation results were reported, as well as incomplete information on the type of transposon carrying this gene, without providing data about the plasmid carrying such gene or its sequence [16].

In our work, by using WGS with a hybrid approach, we were able to determine that *rmtD2* is located on a cointegrated plasmid of 149.5 kb belonging to the incFI(B)/IncFII incompatibility groups. Additionally, we were able to determine that the Tn*21* derivative carrying this structure is a new transposable unit named Tn*7825*. In addition to the 16S-RMTase gene, bracketed by two IS*CR14* (where the second copy, named ISC*R14b*, is proposed to be a chimera due to the recombination between two IS*CRs*), also contains a class 1 integron with a variable region comprising *aacA4*-*bla*_OXA-101_ [16]. Furthermore, it contains genes conferring resistance to quaternary ammonium compounds and sulfonamides (*qacEΔ1*-*sul1*), as well as efflux pumps conferring resistance to chloramphenicol (*floR*) and tetracycline (*tet*). Moreover, as a transposable unit derived from Tn*21*, Tn*7825* includes the mercury resistance operon *merRTPCADE* [17].

Conversely, the presence of *bla*_OXA-101_ was first reported in Argentina in 2010 as part of an integron with the same structure as reported here and obtained from isolates of *E. cloacae*, *C. freundii,* and *E. coli* also resistant to gentamicin and amikacin. However, the presence of methylases was neither explored in such isolates nor in the obtained transconjugants [18].

In a similar study from 2011 by Tijet et al., where the first description of *rmtD2* is mentioned, the sequences deposited in the databases (acc. Nos. HQ401566 and HQ401567) only include the partial sequence of a class 1 integron described as *aacA4*-*bla*_OXA-like_. Based on these two backgrounds, it could be speculated that Tn*7825* or derived structures may have already been disseminated regionally since that period. Neither of the aforementioned reports describes the plasmid incompatibility group that mobilizes these genes.

The presence of 16S-RMTases has often been associated with carbapenemase-producing isolates, which exacerbates the phenomenon of multi-resistance and co-selection [19,20]. In this sense, the remarkable increase in the incidence of carbapenemases observed during the COVID-19 pandemic could partially explain the dramatic growth in 16S-RMTases, as reported by Sellera et al. since 2020 [21].

In this regard, four out of the five isolates reported in our work were also carbapenemase producers. The first of these was obtained in 2017 and consisted of a *K. pneumoniae* ST258 harboring a plasmid encoding *rmtC* and *bla*_NDM-1_. While other reported *K. pneumoniae* isolates carrying RmtC belong to various sequence types, primarily from clonal complexes CC14 and CC11, none of them correspond to ST258, as described in this study [22].

The genetic environment of *rmtC* and *bla*_NDM-1_ described in pUyRmtC117 has been reported in isolates of *P. mirabilis* from Australia and *S. enterica* from the United Kingdom. However, the plasmids described in these studies show variability, unlike in Argentina, where plasmids carrying these resistance mechanisms exhibit a 99% similarity with isolates of *Citrobacter freundii* and *Enterobacter cloacae* (pCFR17394 and pECL17464, respectively) [7,22,23].

Last but not least, from collections obtained in 2022 and 2023 from two different hospitals, we detected three ST307 *K. pneumoniae* isolates harboring *rmtB* and *bla*_NDM-5_. In this regard, although such isolates were recovered in different years and from different hospitals, the analysis of core genome SNPs indicated that they corresponded to the same strain.

*K. pneumoniae* ST307 was proposed as a high-risk clone in 2017 based on its ability to thrive in hospital environments, its capacity to harbor multiple virulence and antibiotic resistance genes, and its capacity for global dissemination [24]. In South America, KPC-producing *K. pneumoniae* ST307 has been described as an emerging clone in countries such as Argentina, Brazil, and Colombia [25,26,27,28]. However, the occurrence of *bla*_NDM-5_ in this high-risk clone has been reported so far in Argentina, and usually in isolates co-harboring *bla*_NDM-5_ and *bla*_KPC-2_ [29,30].

In accordance with González-Espinosa et al., *K. pneumoniae* ST307 readily incorporates different plasmids encoding resistance genes. In this regard, our study describes isolates that harbor several plasmids encoding different ESBL genes such as *bla*_CTX-M-15_, *bla*_CTX-M-8,_ and *bla*_SHV-28_. In Metallo-carbapenemase-producing isolates, the presence of ESBLs rules out the use of one of the available therapeutic options like aztreonam

The association between high-risk clones with multi-drug resistance genetic platforms entails a serious problem for public health on account of constituting an important source for co-selection of antibiotic resistance. In this context, the genetic platform encoding *rmtB* also harbors genes conferring resistance to every available β-lactams, including aztreonam and ceftazidime-avibactam, due to the presence of *bla*_NDM-5_ and *bla*_CTX-M-15_. Moreover, it also confers resistance to every aminoglycoside due to the presence of *rmtB* and *aadA2* (the latter conferring resistance to streptomycin, which is usually unaffected by 16S-RMTases), resistance to trimethoprim-sulfamethoxazole due to *sul1*, *dfrA12,* and *dfrA14*, and also resistance to macrolides on account of the presence of *ermB* and the *mphA-mpxA-mphR* operon.

Although carbapenem resistance in Enterobacterales does not seem to be associated with the administration of macrolide antibiotics, it should be noted that azithromycin has antimicrobial activity against this group of Gram-negative bacteria. Furthermore, this group of antibiotics was one of the most frequently used during the COVID-19 pandemic [31]. In this context, regardless of which of the aforementioned antibiotic groups is employed to treat any given infection (including macrolides in respiratory infections), they will exert a selective pressure that will favor the survival of any microorganism harboring the *bla*_NDM-5_/*rmtB* platform.

While 16S-RMTases are uncommon resistance mechanisms in our country and typically appear as isolated cases, they are beginning to emerge in different healthcare institutions and at unrelated periods. Previously, the *rmtG* methyltransferase had been reported in one-day-old chicks in IncQ plasmids in Uruguay, as well as the occurrence of *rmtC* in clinical isolates of *A. baumannii* [13,14]. The association of these mechanisms with carbapenemases, primarily NDM-1 (the most common carbapenemase in the country) and NDM-5 (the first local report), raises concerns regarding the platforms that harbor them and favor their dissemination. Furthermore, the fact that these platforms resemble those found in the region suggests the potential for geographical spread and trafficking of resistance genes.

Recently, the emergence of multidrug-resistant and pan-resistant microorganisms has created the need to explore new therapeutic approaches to address these types of infections. In this context, the combination of antibiotics with synergistic effects together with bacteriophage therapy appears as a promising tool [32,33]. However, likely due to millions of years of co-evolution between bacteriophages and bacteria, knowledge related to the presence of mechanisms linked to defense (or resistance) to phage infection has recently exploded [34].

In this work, four out of five plasmids encoding 16S-RMTases also encoded resistance mechanisms to bacteriophage infection. Thus, pUYrmtD2 encoded four defense systems against phages (CBASS, the toxin/antitoxin systems involved in programmed cell death Mok-Hok-Sok and PfiAT, and Detox responsible for premature phage lysis). Meanwhile, three plasmids carrying *rmtB* also harbored anti-phage defense mechanisms, including the *mazEF* toxin/antitoxin system and the restriction-modification (RM) system.

In times when bacteriophage treatment is becoming a possibility, the monitoring of the emergence of resistance mechanisms is also a concerning issue, especially when associated with plasmids carrying multidrug resistance to antimicrobials.

Finally, the description and characterization of plasmids such as those mentioned in the present study will allow us to understand the dissemination of resistance mechanisms, and to develop effective strategies to combat the spread of AMR.

## 4. Materials and Methods

### 4.1. Strains

We reviewed the antibiotic susceptibility of 167 previously reported Enterobacterales, searching for isolates displaying simultaneous resistance to gentamicin and amikacin. The identification and susceptibility testing were conducted using the Vitek 2 System (bioMériex, Hazelwood, MO, USA), and the Kirby–Bauer disc-diffusion method, respectively. Antibiotic susceptibility results were interpreted according to the Clinical Laboratory Standard Institute (CLSI) guidelines [35,36,37,38].

American-Type Culture Collections ATCC 25922, ATCC 700603, and ATCC 31488 were used as quality control strains.

### 4.2. 16S rRNA-Methyltransferase and Carbapenemase Detection

Isolates resistant to both gentamicin and amikacin were tested for high-level aminoglycoside resistance, as previously described by Hidalgo et al. [39], with minor modifications. Briefly, 3 µL of an inoculum equivalent to 0.5 McFarland of each isolate was plated on MacConkey-lactose agar plates supplemented with 200 mg/L of amikacin and gentamicin. Growth after 18–28 h of incubation was considered a positive screening result [13]. On the other hand, carbapenemase production was screened for following the CLSI guidelines. Double disk synergy tests between imipenem and meropenem, using ethylenediaminetetraacetic acid (EDTA) and boronic acid, were employed to assess phenotypic carbapenemase production [40].

Molecular detection of 16S-RMTases (*armA*, *rmtA-rmtH*, and *npmA*) and carbapenemase genes (*bla*_KPC_, *bla*_VIM_, *bla*_IMP_, *bla*_NDM_, *bla*_GES_, and *bla*_OXA-48_) was achieved using PCR, and results were confirmed by sequencing the PCR products, as previously described [13,40].

### 4.3. Conjugation Assays

Plasmid transfer was assayed by liquid-phase mating assay; *E. coli* J53-2 (*pro met* Rif^r^ Nal^r^) was used as the recipient strain. Putative transconjugants were selected on Luria-Bertani agar plates supplemented with the appropriate antibiotics, as previously described [40].

### 4.4. Whole Genome Sequencing

Genomic DNA extraction was performed with the NZY microbial gDNA isolation kit, following the manufacturer’s instructions (NZYTech Genes & Enzymes, Lisbon, Portugal). The quality of the extracted DNA samples was measured using a NanoDrop 1000 spectrophotometer (Thermo Fisher, Wilmington, DE, USA); DNA quantification was carried out using a Qubit^®^ 3.0. fluorometer and the dsDNA HS Assay kit (Thermo Fisher Scientific, Waltham, MA, USA).

Illumina libraries were prepared using the Nextera XT DNA Library Prep Kit along with the Nextera XT Index kit (Illumina Inc., San Diego, CA, USA) and sequenced with an Illumina MiniSeq device and a MiniSeq High.output reagent kit (Illumina Inc., San Diego, CA, USA), following a 2 × 151 bp paired-end approach.

On the other hand, all isolates were also subjected to long-read sequencing using Oxford Nanopore Technologies, Oxford, UK. In this regard, nanopore libraries were prepared with the Rapid Barcoding Kit SQK-RBK004 following the manufacturer’s guidelines. Libraries were subsequently sequenced for 8 h in a MinION MK1B device using an R9.4.1 flow cell (Oxford Nanopore Technologies). Later, base-calling of long reads was performed using Guppy standalone version 6.5.7 with the High-accuracy model.

Illumina-generated short reads were filtered with the Fastp software v0.23.2 [41] and assembled with SPAdes v3.15 [42]; conversely, Nanopore-generated reads were filtered using Filtlong v0. 2.1 (https://github.com/rrwick/Filtlong, accessed on 1 March 2024), removing those reads < 2000 bp and displaying a mean quality score < 93; the quality of the trimmed reads was assessed with NanoPlot v1.33.1 [43,44].

Finally, de novo hybrid genome assembly (using short and long reads), was carried out using the Unicycler software v0.4.8, following a conservative approach [44].

### 4.5. In Silico Analysis

The occurrence of antimicrobial resistance genes was predicted using both the Comprehensive Antibiotic Resistance Database (CARD) (https://card.mcmaster.ca/, accessed on 1 April 2024) and the Resfinder 4.1 online service [45]. In both instances, stringent criteria were used to attain high coverage and nucleotide identity. On the other hand, the presence of putative plasmid incompatibility groups was predicted using the PlasmidFinder 2.1 suite (minimum identity 95%, minimum coverage 60%). Finally, multilocus sequence typing of the sequenced genomes was determined using the MLST 2.0 online database. MLST 2.0 and PlasmidFinder 2.1 are available on the Center for Genomic Epidemiology webpage (https://www.genomicepidemiology.org/services/, accessed on 1 April 2024).

The determination of defense systems was conducted using the DefenseFinder web service (https://defensefinder.mdmlab.fr/, accessed on 1 April 2024) in plasmids containing HLAR mechanisms [46,47,48].

Genomes were annotated using Prokka v3.2.1, and plasmid annotation files were manually curated with Artemis software v17.0.1 [49,50].

Pan-genome analysis and identification of putative orthologous genes were using the ROARY package v3.13.0 [51]; conversely, mutation assessment was performed with the breseq/gdtools pipeline v0.37.1 [52].

## Figures and Tables

**Table 1:** Characteristics of the HLAR Enterobacterales isolates analyzed in this study, including isolation date, sample, 16S rRNA-methyltransferase, antimicrobial susceptibility profiles, plasmids, and antibiotic resistance genes.

Strain	Isolation Date and Hospital	Sample	Species and Sequence Type	Rmt/CARB	MIC (mg/L)															Incompatibility Group and Plasmid Size (bp)	Antibiotic Resistance Genes
					AK	GM	PTZ	CTX	CAZ	CZA	FEP	ATM	IPM	MEM	ETP	CIP	SXT	COL	TIG		
UH_B1	08/2022 H1	Blood	*K. pneumoniae* ST307	RmtB/NDM-5	>32	>8	>64	>32	>32	>256	16	>16	4	2	>2	>2	>2	≤1	≤0.5	IncR (55,925 bp)	*dfrA12*, *aadA2*, *sul1*, *bla*_NDM-5_, *rmtB*, *bla*_TEM-1B_, *bla*_CTX-M-15_, *dfrA14*, *mph(A)*, *ermB*, *qacEΔ1*
																				IncFIB(K)/IncFII (217,500 bp)	*sul2*, *aph(3″)-Ib*, *aph(6)-Id*, *bla*_TEM-1B_
UH_B2	08/2022 H2	Urine	*K. pneumoniae* ST307	RmtB/NDM-5	>32	>8	>64	>32	>32	>256	16	>16	2	2	>2	>2	>2	≤1	≤0.5	IncR (55,916 bp)	*dfrA12*, *aadA2*, *sul1*, *bla*_NDM-5_, *rmtB*, *bla*_TEM-1B_, *bla*_CTX-M-15_, *dfrA14*, *mph(A)*, *ermB*, *qacEΔ1*
																				IncFIB(K)/IncFII (217,500 bp)	*sul2*, *aph(3″)-Ib*, *aph(6)-Id*, *bla*_TEM-1B_
																				IncM1 (68,728 bp)	*bla*_CTX-M-8_, *bla*_TEM-1B_, *qnrE1*
																				IncFIB(K)/IncFII (129,906 bp)	*qacEΔ1*, *mph(A)*, *catB3*, *sul1*, *aac(6′)-Ib-cr*, *aadA2*, *aac(3)-IIa*, *bla*_TEM-1B_, *bla*_SHV-28_, *bla*_OXA-1_, *aac(6′)-Ib-cr*, *dfrA12*
UH_B3	09/2023 H1	Abscess	*K. pneumoniae* ST307	RmtB/NDM-5	>32	>8	>64	>32	>32	>256	>16	>16	4	8	>2	>2	>2	≤1	≤0.5	IncR (61,310 bp)	*dfrA12*, *aadA2*, *sul1*, *bla*_NDM-5_, *rmtB*, *bla*_TEM-1B_, *bla*_CTX-M-15_, *dfrA14*, *mph(A)*, *ermB*, *qacEΔ1*
																				IncFIB(K)/IncFII (212,501 bp)	*dfrA14*, *mph(A)*, *bla*_CTX-M-15_, *bla*_TEM-1B_, *aph(6)-Id*, *aph(3″)-Ib*, *sul2*
UH_C1	11/2017 H3	Urine	*K. pneumoniae* ST258	RmtC/NDM-1	>32	>8	>64	>32	>32	>256	>16	>16	>8	16	>2	>2	≤2	≤1	≤0.5	IncC (138,998 bp)	*bla*_CMY-6_, *aac(6′)-Ib*, *sul1*, *rmtC*, *bla*_NDM-1_
																				ColRNAI (15,271 bp)	*aac(6′)-Ib*
																				IncFIB(K)/IncFII (137,364 bp)	*mph(A)*
UH_D1	12/2010 H1	Rectal colonization	*E. cloacae* ST88	RmtD/-	>32	>8	>64	>32	32	≤0.5	≤2	>16	≤0.5	≤1	2	>2	>2	≤1	≤0.5	IncFIB(K)/IncFII (149,562 bp)	*sul1*, *tet(G)*, *floR*, *rmtD2*, *sul1*, *bla*_OXA-101_
																				IncFII (130,409 bp)	*bla*_TEM-258_, *aac(3)-IIa*, *bla*_TEM-1a_

MIC: Minimum Inhibitory Concentration. AK: amikacin GM: gentamicin PTZ: piperacillin/tazobactam CTX: cefotaxime CAZ: ceftazidime CZA: ceftazidime-avibactam FEP: cefepime ATM: aztreonam IPM: imipenem MEM: meropenem ETP: ertapenem CIP: ciprofloxacin SXT: trimethoprim/sulfamethoxazole COL: colistin TIG: tigecycline. RMT/CARB: 16S-RMTase/carbapenemase.

## Data Availability

Whole-genome sequence data were deposited in the GenBank database under BioProject acc. No. PRJNA1131454 and the plasmids, along with *bla*_TEM-258_, were assigned with the acc. Nos. PQ203016, PQ212794, PQ203015, PQ203988, PQ122004, PQ328755, and PQ362078.
